# Detroit Veterinary College

**Published:** 1896-09

**Authors:** 


### ONTARIO VETERINARY COLLEGE.

### DETROIT VETERINARY COLLEGE.

THE Veterinary Department of the Detroit College of Medicine will open its course on September 23d and continue for six months. The school still adheres to the two-year plan. The new buildings are expected to be ready for occupancy this year.

The resignations of Professors Brenton and McEachran necessitated changes in the course of instruction. J. D. Rutherford, M.D.V.S., will fill the chairs of anatomy, principles and practice of veterinary surgery, and obstetrics. M. L. High, M.D.V.S., graduate of the Ontario Veterinary College, Class of 1877, will fill the chair of materia medica and therapeutics. H. Rutherford, D.S., D.V.S., graduate of the Detroit Veterinary College, Class of 1895, has been appointed professor of principles and practice of veterinary medicine. But three veterinarians are noted on the faculty. They again announce an early adoption of a three-year course.

